# Hepatoprotective, Lipid-Lowering and Antioxidant Effects of Mangaba Powder (*Hancornia speciosa*) Administered to Rats Fed a High-Fat Diet

**DOI:** 10.3390/foods13233773

**Published:** 2024-11-25

**Authors:** Bernadete de Lourdes de Araújo Silva, Margarida Angélica da Silva Vasconcelos, Kamila Sabino Batista, Marcos dos Santos Lima, Fabiane Rabelo da Costa Batista, Hassler Clementino Cavalcante, Lydiane de Lima Tavares Toscano, Alexandre Sérgio Silva, Aline Barbosa D’Oliveira, Adriano Francisco Alves, Jailane de Souza Aquino

**Affiliations:** 1 Nutrition Department, Universidade Federal de Pernambuco, Recife 50670-901, Brazil; bernadete.araujo2@academico.ufpb.br (B.d.L.d.A.S.); margarida.vasconcelos@ufpe.br (M.A.d.S.V.); 2 Nutrition Department, Universidade Federal da Paraíba, João Pessoa 58051-900, Brazil; hasslerrcavalcante@gmail.com (H.C.C.); lyditavares@hotmail.com (L.d.L.T.T.); alexandre.sergio@ccs.ufpb.br (A.S.S.); 3 Experimental Nutrition Laboratory, Universidade Federal da Paraíba, João Pessoa 58051-900, Brazil; kamila.sabino@outlook.com (K.S.B.); allineolliveira99@gmail.com (A.B.D.); 4 Instituto Nacional do Semiárido, Campina Grande 58434-700, Brazil; fabiane.costa@insa.gov.br; 5 Department of Food Technology, Federal Institute of the Sertão of Pernambuco (IFSertãoPE), Petrolina 56316-686, Brazil; 6 Department of Physiology and Pathology, Universidade Federal da Paraíba, João Pessoa 58051-900, Brazil; adrianofalves@ccs.ufpb.br

**Keywords:** antioxidants, Apocynaceae family, dehydrated fruits, dyslipidemia, hyperlipidic diet

## Abstract

The aim of this study was to evaluate the potential effects of administering mangaba powder on liver function and somatic, oxidative and lipid metabolism parameters in rats fed a high-fat diet. Prepared mangaba powder has important amounts of phenolic compounds, vitamin C, dietary fiber and oligosaccharides. A total of 32 adult Wistar rats were initially randomized into two groups for the biological assay: normal-fat (NF, n = 16) and high-fat (HF, n = 16) diets for 21 days. These rats were subsequently subdivided into four groups: NF (n = 8), HF (n = 8), normal-fat diet with mangaba powder administration (NFMG, n = 8) and high-fat diet with mangaba powder administration (HFMG, n = 8). The treatment with mangaba powder (400 mg/kg) lasted an additional 28 days. Compared to the HF rats, the HFMG rats showed an 8% reduction in the body mass index. Treatment with mangaba reduced the serum cholesterol by 18%, as well as the hepatic deposition of triacylglycerides by 26% and cholesterol by 25%, in addition to increasing bile acid synthesis by 77% in this organ. Mangaba powder consumption attenuated the degree of hepatic steatosis, reduced lipid peroxidation and increased the serum and hepatic antioxidant capacity in HFMG rats. These results show that the consumption of mangaba powder had lipid-lowering, hepatoprotective and antioxidant effects, especially in HFMG rats, which may be associated with an additive and synergistic action between the bioactive compounds present in the product.

## 1. Introduction

Dyslipidemia is a clinical condition characterized by increased plasma total cholesterol, low-density lipoprotein (LDL), and triacylglycerides, and low levels of high-density lipoprotein (HDL) [1], and it may be related to oxidative damage in the organism [2], non-alcoholic fatty liver disease (NAFLD) in which there is an accumulation of fat in the liver and atherosclerosis [3]. This clinical condition with a multifactorial cause is considered a risk factor for cardiovascular diseases, which represent one of the major causes of death worldwide [4].

In this sense, animal models consuming a high-fat diet rich in cholesterol and saturated and trans fatty acids have been used to mimic these diseases in humans to demonstrate alternatives for prevention and treatment [5,6].

Dietary interventions are especially proposed for treating dyslipidemia and other cardiovascular clinical conditions. The consumption of at least 400 g/day of fruits and other vegetables, except those rich in starch, has been recommended as a strategy for preventing chronic non-communicable diseases and their comorbidities [7]. These recommendations are related to the diversity of bioactive compounds existing in these foods, particularly in fruits, which have compounds that perform important biological activities in the body.

Previous in vivo studies have demonstrated that freeze-dried tropical fruits and their by-products, such as acerola (*Malphigia ermaginata*), Malay apple (*Syzygium malaccense*) and cajá (*Spondias mombin*), possess nutraceutical properties, including antioxidant and lipid-lowering properties and the reversion of hepatic steatosis in rats fed a high-fat diet [8,9,10]. These fruits and their by-products are rich in bioactive compounds as well as mangaba (*Hancornia speciosa*). Mangaba has recognized in vitro antioxidant activity, a high content of ascorbic acid, phenolic compounds, and carotenoids [11], in addition to minerals as the main bioactive compounds that can have antioxidant, anti-cancer and cardioprotective effects [12,13,14]. Mangaba additionally has soluble and insoluble fibers, and these components are associated with the ability to modulate food digestion, nutrient absorption and metabolism [15].

Despite Brazil being the second largest producer of tropical fruits in the world [16], many fruits are still underexploited, either by the food industry or the pharmaceutical industry, with a focus on herbal and nutraceuticals. Approximately 47% of Brazilian fruit production is destined for fresh consumption, while 53% is intended for the processing industry [17]. Among the various Brazilian tropical fruits, mangaba is highly perishable and can undergo rapid enzymatic browning when consumed in natura or when the pulp is processed [18].

In this context, lyophilization, or freeze drying, is one of the most effective technological processes for preserving the bioactive compounds in fruits and reducing their perishability [19,20]. In this sense, the powdered fruit market is expanding due to the advantages of greater conservation of nutraceutical properties, convenience in consumption, storage, and greater availability for commercialization due to increased shelf-life [19]. Furthermore, lyophilization can be optimized by using a drying adjuvant such as amphiphilic whey protein to encapsulate and protect the bioactive molecules in various food matrices; eliminate the negative interference of fruit stickiness and caking phenomena, especially seen during powder production in mangaba; and prevent enzymatic browning of this fruit [18,20,21]. Despite this, to date, no study has used whey protein as a drying adjuvant in the production of mangaba powder and evaluated the effects of this product on the antioxidant status and lipid metabolism in an animal model.

Considering that mangaba is still a little-explored seasonal tropical fruit by the agroindustry sector and that it presents several bioactive compounds evaluated in vitro, with promising beneficial health properties, the objective of this study was to characterize the main bioactive compounds of lyophilized mangaba with added whey protein and to evaluate the potential effects of its administration on the liver function and somatic, oxidative and lipid metabolism parameters of rats fed a normal- or high-fat diet.

## 2. Materials and Methods

### 2.1. Acquisition and Preparation of Mangaba Powder (Hancornia speciosa)

Mangaba fruits (*Hancornia speciosa*) were collected in the Caatinga biome of north-eastern Brazil (latitude 06°41′18″ S, longitude 34°56′09″ W; altitude 2 m). After collection, the fruits were selected by skin color (yellow with red pigments), characteristic aroma, being without visible mechanical damage, and being free of parasites and fungi. They were transported to the laboratory in polyethylene boxes, then washed and sanitized in running water and a 50 ppm sodium hypochlorite solution for 15 min. Pulping was performed in a semi-industrial machine (model DES-60/1, Braesi, Caxias do Sul, Brazil). Concentrated whey protein (WPC 85% protein, Uninutri, Juazeiro do Norte, Brazil) was added to the fresh pulp at a concentration of 10% (*w*/*w*) as a drying aid.

The sample was dehydrated in a benchtop lyophilizer (model L101, Liobras, Campinas, Brazil) for 72 h at −40 °C with a constant speed of 1 mm/h, a vacuum of 0.5 mm Hg and a final pressure of 0.05 mmHg. After lyophilization, the product was ground in a food processor (Mixer Philips Walita, Itaipava, Brazil), placed in glass containers with lids, protected from light and stored at −18 °C. The method for obtaining mangaba powder was selected based on a pilot study, which was previously carried out to determine the drying conditions and the best adjuvant concentration to protect the bioactive compounds in the product. In this pilot study, it was observed that the freeze-drying process could not be satisfactorily applied to mangaba pulp without a drying adjuvant, as it caused a reduction in antioxidant activity and some bioactive components.

### 2.2. Analyses of Bioactive Compounds and Nutritional Parameters of Mangaba Powder

The total carotenoids was determined by the spectrophotometric method. Thus, 18 mL of pure acetone were initially added to 0.5 g of mangaba powder and read in a spectrophotometer (model Genesys 10S UV-Vis, Thermo Fisher Scientific, Inc., Madison, WI, USA) at wavelengths of 470 nm, 645 nm and 662 nm, in the absence of light and at a temperature of 25 ± 1 °C to determine the total carotenoids [22]. The ascorbic acid content was colorimetrically determined in a spectrophotometer at 518 nm [23]. The total phenolic compounds was determined at 765 nm in a spectrophotometer (model Genesys 10S UV-Vis, Thermo Fisher Scientific, Inc., Madison, WI, USA).

The phenolic [24] and oligosaccharide [25] profiles were quantified using high-performance liquid chromatography (HPLC) with an Agilent 1260 system coupled to a refractive index detector (model G1362A, Agilent Technologies, Inc. Santa Clara, CA, USA) under the determined analytical conditions. Previously, aqueous and methanolic extracts of mangaba powder were prepared for oligosaccharide and phenolic compound quantification, respectively. The aqueous extracts were prepared by mixing 2 g of mangaba powder and 10 mL of ultrapure water, followed by homogenization for 10 min, centrifugation (3500× *g*, 10 min, 24 °C) and filtering the supernatant through a 0.45 µm nylon membrane (Millex Millipore, Barueri, Brazil). In turn, the methanolic extract that was used to carry out the analysis of phenolic compounds by HPLC was prepared with 2 g of mangaba powder added to 10 mL of methanol:ultrapure water (70:30, *v*/*v*), followed by sonication (40 kHz/30 min at 27 °C) and centrifugation (3500× *g*, 10 min, 24 °C). These procedures were repeated twice, and the collected supernatants were mixed and filtered (0.45 µm membrane pore size).

Antioxidant activity was determined by the free radical scavenging method with DPPH (1,1 diphenyl-2-picrylhydrazyl) and ABTS (2.2′-azino-bis 3-ethylbenzothiazoline-6-sulfonic acid). The aqueous extract was prepared with 1 g of mangaba powder diluted in 10 mL of ultrapure water. DPPH (Sigma-Aldrich, St. Louis, MO, USA) 1.0 mM solution (2.9 mL) was mixed with 100 µL aqueous extracts and ABTS (Sigma-Aldrich, St. Louis, MO, USA) solution (3 mL) was mixed with 30 µL aqueous extracts, with the respective absorbance readings at 517 nm and 754 nm [26]. Then, the contents of total dietary fiber, insoluble dietary fiber and soluble dietary fiber were determined using the enzymatic-gravimetric method with the alpha-amylase, protease and amyloglucosity enzymes (Megazyme, Ltd., Bray, Ireland) [23,27] (Table S1).

### 2.3. Experimental Design

A total of 32 Wistar rats with an initial age of approximately 60 days and a body weight between 245 and 295 g were used in the experiment. The animals were kept in collective cages (four animals/cage) in an environment with a temperature of 21 °C ± 1 °C, relative humidity between 50 and 55%, with a 12 h light/dark cycle. The experimental protocol was approved by the Animal Use Ethics Committee of the Federal University of Pernambuco (UFPE), Recife, Brazil, under number 23076.010214/2018-43. The experimental protocol followed the Animal Research: Reporting of In Vivo Experiments: the ARRIVE Guidelines [28].

After the one-week acclimation period, the Wistar rats were randomized into two groups, maintaining a similar initial weight average between them: the normal-fat group (NF, n = 16), which received standard AIN-93M diet with soybean oil as a source of fat (4%), and the high-fat group (HF, n = 16) fed a high-fat diet (Rhoster, Araçoiba da Serra, Brazil) containing 6% lard, 5% non-hydrolyzed vegetable fat, 3% soy oil and 1% cholesterol as lipid sources (Table S2). The fatty acid composition of the diets used is shown in Table S3.

The administration of the diets (normal fat or high fat) took place for three weeks and the blood of the NF and HF rats was collected after this period via the orbital plexus for diagnosis of changes in the lipid profile by biochemical analysis. Then, the rats were relocated into four groups: NF (n = 8) and HF (n = 8), which continued with their respective diets and started to receive gavage with 2 mL of saline solution during the following four weeks; and the normal-fat diet and mangaba powder administration (NFMG, n = 8) and high-fat diet and mangaba powder administration (HFMG, n = 8) groups, which started to receive gavage with mangaba powder (400 mg/kg of body weight diluted in 1.6% *w*/*v* of saline solution) during the same period. The dietary intake and body weight were assessed in all the groups every week. The weekly energy intake (kcal) was calculated based on the dietary intake (g) according to the energy value offered by each diet [5].

To select the dose of mangaba powder administered to the rats, we conducted a pilot study with three different doses (100, 200 and 400 mg/kg), and 400 mg/kg showed the best results in the lipid profile of the rats. We also considered previously reported results concerning hypolipidemic effects and liver health in rats fed a high-fat diet based on consumption of other tropical fruits and their by-products at a dose of 400 mg/kg [5,8,9,10].

### 2.4. Somatic Parameters and Euthanasia

After seven weeks of the experiment, which corresponded to the pre-treatment and treatment with mangaba powder, the animals were fasted for 8 h and anesthetized with intraperitoneal injection of ketamine hydrochloride (75 mg/kg) and xylazine (5 mg/kg) to measure the somatic parameters using an inelastic measuring tape. The waist circumference, chest circumference and body length were measured. The body mass index was calculated by dividing the body weight (g) by the length squared (cm^2^), and the Lee index was obtained through the ratio between the cubic root of the body weight (g) by the length (cm) of the animal [29].

Euthanasia was performed via the animals being anesthetized using the cardiac puncture technique for blood collection, which was used to obtain serum via centrifugation (1.040× *g*/10 min) for biochemical analysis. Adipose tissue was collected for weighing and calculation of the adiposity index: [body fat weight (epididymal + visceral + retroperitoneal)/body weight] × 100 [30].

The liver (left lobe), intestine (colon) and adipose tissue were collected, washed with saline solution, weighed and stored in 10% buffered formalin for histological analysis. Part of the liver samples were kept under freezing conditions (−20 °C) for the analysis of the oxidative parameters, cholesterol, triacylglycerides and bile acids.

### 2.5. Determination of Lipid Profile, Aminotransferases and Calculation of Indices Related to Cardiovascular Health

The serum aspartate aminotransferase (AST) and alanine aminotransferase (ALT) enzymes were determined using commercial kits (Labtest^®^, Belo Horizonte, Brazil), following the label instructions with reading at 340 nm in an automatic analyzer (model LabMax 240 Premium, Belo Horizonte, Brazil). The serum triacylglycerides, total cholesterol, HDL and LDL concentrations were also measured using commercial kits (Labtest^®^, Belo Horizonte, Brazil). These analyses followed the manufacturer’s recommendations and were analyzed with the same automatic analyzer at 505 nm (triacylglycerides), 500 nm (total cholesterol), 600 nm (HDL) and 546 nm (LDL). The very-low-density lipoprotein (VLDL) values were calculated using the following equation: VLDL = triacylglycerides/5. The atherogenic index, the coronary risk index and the cardiovascular risk index were calculated according to the following equations: atherogenic index = LDL/HDL; coronary risk index = total cholesterol/HDL and cardiovascular risk index = triacylglycerides/HDL [31].

### 2.6. Hepatic and Fecal Cholesterol, Triacylglycerides and Total Bile Acids

Feces was collected in the last week of the experiment for three consecutive days for the total cholesterol, triacylglycerides and total bile acids analyses. Samples for the total cholesterol and triacylglycerides analysis in the liver and feces were macerated, and then the liver and fecal lipids were extracted according to the methodology developed by Folch, Less and Stanley [32]. An aliquot (5 mL) of fat was collected in sterile tubes to determine these parameters. Next, the total cholesterol and triacylglycerides quantification was performed with a commercial kit (Labtest^®^, Belo Horizonte, Brazil), as described in Section 2.6, with reading at 500 nm for the total cholesterol and 505 nm for the triacylglycerides, performed in a spectrophotometer (model UV-Visible Shimadzu 1650-PC, Tokyo, Japan).

The hepatic and fecal total bile acids were determined by the colorimetric reaction of ethanol extracts obtained by the Folch, Less and Stanley [32] method of the upper layer with a 2.5% phosphomolybdic acid solution, followed by a spectrophotometer reading at 690 nm. The total bile acids content of the samples was determined according to the sodium glycocholate standard curve (Sigma-Aldrich, Darmstadt, Germany) [33].

### 2.7. Hepatic and Serum Oxidative Parameters

Lipid peroxidation was evaluated by measuring the thiobarbituric acid reactive substances and expressed as the malondialdehyde content [34], and the total antioxidant capacity was measured by the free radical scavenging activity according to the DPPH method [35]. The total antioxidant capacity results were expressed as a percentage of the antioxidant activity and calculated using the following equation: antioxidant activity (%) = 100 − [DPPH•R] t/[DPPH•R]B 100), in which [DPPH•R]t and [DPPH•R]B correspond to the DPPH free radical concentrations remaining after 30 min evaluated in the sample (t) and in the blank (B). Both reactions were read in an ultraviolet spectrophotometer (model UV-Visible Shimadzu 1650-PC, Tokyo, Japan) at wavelengths of 535 nm (malondialdehyde) and 515 nm (total antioxidant capacity), respectively.

### 2.8. Histological Analyses of Liver, Intestine and Adipose Tissue

Liver (left lobe), intestine (colon) and adipose tissue fragments were stored in 10% buffered formalin for 48 h and subjected to routine histological processing. To do so, the samples were dehydrated in increasing ethanol concentrations, clarified in xylol, embedded in paraffin and sectioned in a microtome, obtaining 4 µm thick slices. The sections were stained with hematoxylin–eosin and later analyzed in a light microscope (Motic BA 200, Santa Monica, CA, USA) under crescent objectives.

The hepatic steatosis degree was determined by semi-quantitative analysis using a scale from 0 to 5, where 0–1 indicates an animal without lesions; 1–2 indicates a discrete lesion distribution (light focal); 2–3 indicates a moderate distribution (pronounced focal to diffuse light) and 3–4 indicates an acute lesion (sharp multifocal to pronounced diffuse) [36]. Next, the number of adipocytes per field was analyzed from the adipose tissue images (epididymal + visceral + retroperitoneal) using the Motic Images Plus 2.0 software program (Motic Asia, Ltd., Kowloon, Hong Kong), and the average area (µm^2^) of 100 adipocytes per sample was also calculated [37].

### 2.9. Statistical Analyses

Data from the NF and HF initial groups (n = 16 rats each) did present a normal distribution (Shapiro–Wilk test) or homogeneity of variance (Levene’s test), and the other four groups (n = 8 rats each) did not present a normal distribution or homogeneity of variance; therefore, parametric and non-parametric tests were chosen to compare the variables, respectively. Student’s *t*-test was applied to compare a variable between the NF and HF, and the Kruskal–Wallis and post hoc tests were applied between the four study groups (NF, NFMG, HF and HFMG). Tukey’s test was applied when a significant difference occurred. The results were expressed as the median and percentiles (25% and 75%). The analyses were performed using the SigmaPlot 12.5 program (Systat Software Inc., San Jose, CA, USA), considering a significance level of 5% (*p* ≤ 0.05). A correlation matrix was constructed, and Spearman’s correlation coefficient (*p*) measured the association between two variables, considering *p* ≤ 0.05 for a significant correlation. The direction of the correlation (positive or negative) was classified as moderate strength (≥0.6<0.8) or strong strength (˃0.8) [38].

## 3. Results

### 3.1. Somatic Parameters and Monitoring Body Weight and Dietary, Lipid and Energy Intake

**Figure 1:**
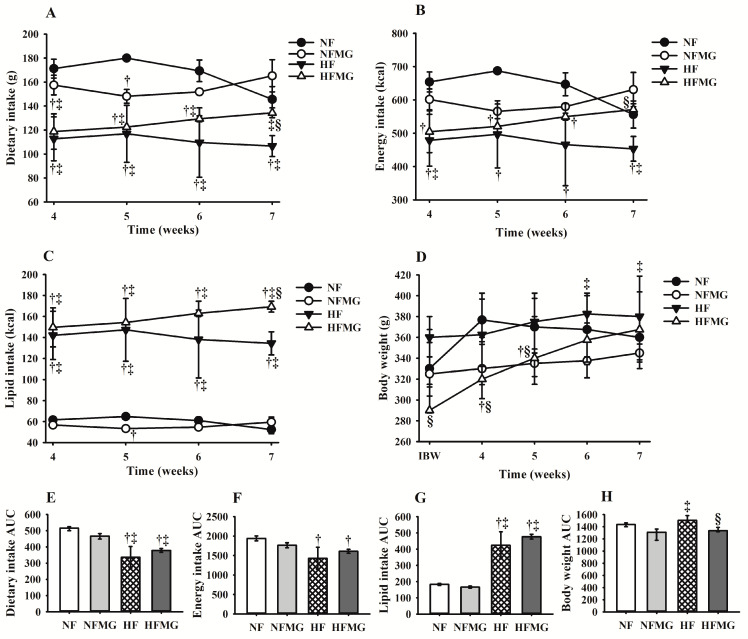
Dietary intake (**A**), energy intake (**B**), lipid intake (**C**), body weight (**D**) and areas under the curve (**E**–**H**) in rats fed normal- or high-fat diets and treated or not with mangaba powder. NF: rats fed a normal-fat diet (n = 8); NFMG: rats fed a normal-fat diet with administration mangaba (n = 8); HF: rats fed a high-fat diet (n = 8); HFMG: rats fed a high-fat diet with administration mangaba (n = 8). AUC, area under the curve; IBW, initial body weight. † significant difference compared with NF; ‡ significant difference compared with NFMG; § significant difference compared with HF. Values are median to the 25th–75th percentiles (Kruskal–Wallis test and Tukey’s post hoc test, *p* ≤ 0.05), n = 8.

The dietary intake and energy intake were lower and the lipid consumption was higher in rats fed a high-fat diet from the second week of the pre-treatment period when compared to rats that consumed a normolipidic diet (*p* ≤ 0.05) (Figure S1). This difference in the consumption pattern between the rats fed a normal-fat diet (NF and NFMG) and a high-fat diet (HF and HFMG) was maintained during the treatment period (Figure 1A–C). Despite the mangaba powder administration causing an increase in dietary and lipid consumption in HFMG rats compared to HF (*p* ≤ 0.05), the body weight of NFMG and HFMG rats in week 7 was lower compared with HF rats (*p* ≤ 0.05) (Figure 1D,H).

At the end of the experiment, the high-fat diet caused an increase in the adiposity index (NF vs. HF, *p* ≤ 0.05), while treatment with mangaba powder was able to reduce the body mass index by 8% (HF vs. HFMG, *p* ≤ 0.05) (Table 1). The liver and adipose tissue of animals in the HF and HFMG had increased weight compared to the NF and NFMG rats (*p* ≤ 0.05) (Table 1).

### 3.2. Effect of Mangaba Powder on Lipid Parameters, Indices Related to Cardiovascular Health and Aminotransferases

**Figure 2:**
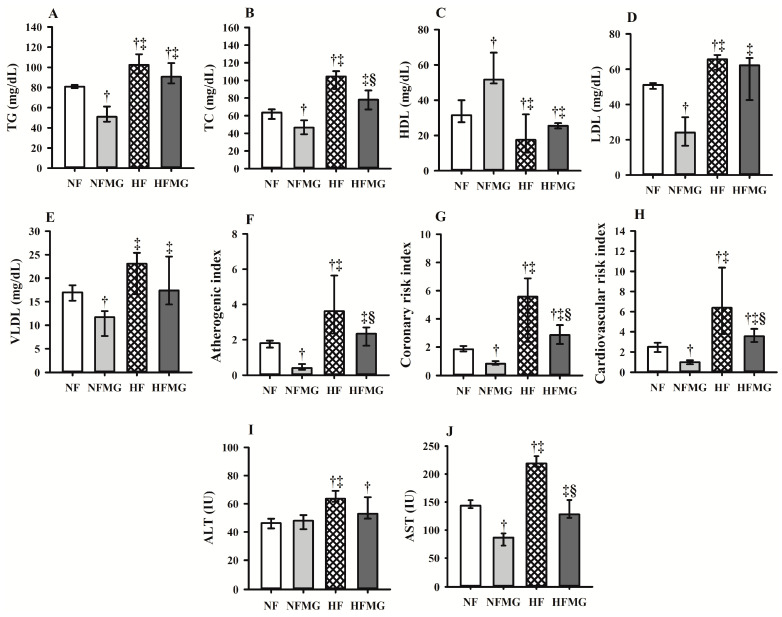
Final lipid profile (**A**–**E**), cardiovascular risk index (**F**–**H**) and transaminases (**I**,**J**) of rats fed normal- or high-fat diets after treatment with mangaba powder. NF: rats fed a normal-fat diet (n = 8); NFMG: rats fed a normal-fat diet with administration mangaba (n = 8); HF: rats fed a high-fat diet (n = 8); HFMG: rats fed a high-fat diet with administration mangaba (n = 8). AST, aspartate aminotransferases; ALT, alanine aminotransferases; TG, triacylglycerides; TC, total cholesterol; LDL, low-density lipoprotein; VLDL, very-low-density lipoprotein; HDL, high-density lipoprotein; IU, international unit. † significant difference compared with NF; ‡ significant difference compared with NFMG; § significant difference compared with HF. Values are median to the 25th–75th percentiles (Kruskal–Wallis test and Tukey’s post hoc test, *p* ≤ 0.05), n = 8.

The HF rats had lower serum HDL levels and increased total cholesterol and LDL levels at the end of the third week of pre-treatment compared to the NF rats (*p* ≤ 0.05) (Figure S2), confirming that the offered high-fat diet was effective in inducing changes in the serum lipid profile in rats. Consumption of mangaba powder reduced the total cholesterol levels (Figure 2B) of the NFMG and HFMG rats, as well as the triacylglycerides (Figure 2A), LDL (Figure 2D), and VLDL (Figure 2E) levels of the NFMG rats at the end of the treatment period (*p* ≤ 0.05), in addition to increasing the HDL levels in these rats (Figure 2C).

The HDL level showed a significant negative correlation with the triacylglycerides (*p* = −0.79), total cholesterol (*p* = −0.74), LDL (*p* = −0.6), atherogenic index (*p* = −0.91), coronary risk index (*p* = −0.91) and cardiovascular risk index (*p* = −0.97) (*p* ≤ 0.05) (Figure S3). The liver weight was positively correlated with the serum triacylglycerides (*p* = 0.72; *p* ≤ 0.001), total cholesterol (*p* = 0.67; *p* ≤ 0.001), and LDL levels (*p* = 0.53; *p* ≤ 0.01), and it had a negative correlation with the serum HDL (*p* = −0.57; *p* ≤ 0.01) (Figure S3), partially indicating impairment of this organ in lipid metabolism.

Treatment with mangaba powder reduced the serum AST enzyme values in the HFMG rats (*p* ≤ 0.05), but there was no significant reduction in the serum ALT enzyme in this group compared to the HF rats (not significant—n.s.) (Figure 2I,J). However, both aminotransferases showed a significant positive correlation (*p* ≤ 0.01) with the atherogenic index (ALT: *p* = 0.69; AST: *p* = 0.68), coronary risk index (ALT: *p* = 0.65; AST: *p* = 0.69) and cardiovascular risk index (ALT: *p* = 0.67; AST: *p* = 0.62) (Figure S3), showing that higher ALT and AST levels negatively influence cardiovascular health.

### 3.3. Triacylglycerides, Total Cholesterol and Total Bile Acids in Feces and Liver

Consumption of mangaba powder increased the fecal triacylglycerides excretion and reduced the hepatic triacylglyceride levels in the HFMG rats compared to the HF rats (*p* ≤ 0.05) (Table 2). The rats’ body weight had a significant negative correlation with the fecal triacylglycerides (*p* = −0.75; *p* ≤ 0.05) and a positive correlation with the hepatic triacylglycerides (*p* = 0.77; *p* ≤ 0.01) (Figure S3).

The treatment with mangaba powder did not influence the fecal total cholesterol excretion in the treated groups (NFMG and HFMG) when compared to their respective control groups (n.s.); however, it reduced the liver total cholesterol in the HFMG rats (*p* ≤ 0.05), which is in line with the results of the lipid profile and may indicate less progression to cardiovascular disease. The NFMG and HFMG rats exhibited increased total bile acid production in the liver compared to their respective controls (*p* ≤ 0.05), but there was no increase in the fecal total bile acid excretion in these groups (n.s.).

### 3.4. Malondialdehyde and Total Antioxidant Capacity

High-fat diet intake caused increased lipid peroxidation in both the serum and liver, and it reduced the liver total antioxidant capacity (DG vs. HG) (*p* ≤ 0.05). Treatment with mangaba powder reduced the serum and liver lipid peroxidation caused by high-fat diet intake (HFMG vs. HF); in addition to increasing the serum and liver total antioxidant capacity, either in rats fed a normal diet (NFMG vs. NF) or high-fat diet (HFMG vs. HF) rats (*p* ≤ 0.05) (Table 3). It is observed that the liver total bile acid levels were positively correlated with the total antioxidant capacity (*p* = 0.87; *p* < 0.001) and negatively correlated with the hepatic malondialdehyde biomarker (*p* = −0.76; *p* < 0.01) (Figure S3).

### 3.5. Effects of Mangaba Powder Treatment on Liver, Intestine and Adipose Tissue Histology

**Figure 3:**
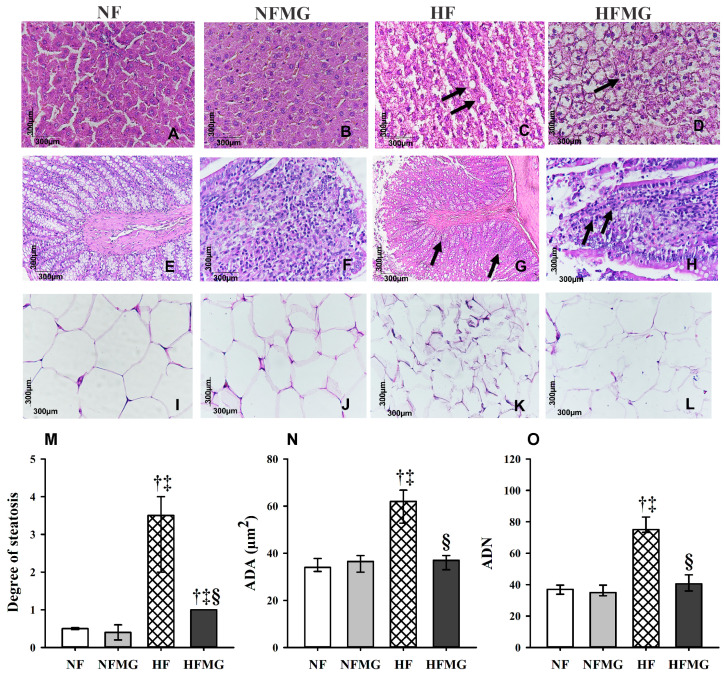
Histological analysis and morphometry of the liver, intestine and adipose tissue of rats fed normal- or high-fat diets after the treatment period with mangaba powder. (**A**): NF group (liver); (**B**): HF group (liver); (**C**): NFMG group (liver); (**D**): HFMG group (liver); (**E**): NF group (intestine); (**F**): HF group (intestine) (**G**): NFMG group (intestine); (**H**): HFMG group (intestine); (**I**): NF group (adipose tissue); (**J**): HF group (adipose tissue); (**K**): group NFMG group (adipose tissue); and (**L**): HFMG group (adipose tissue). Arrows indicate hepatocytes with clear cytoplasm and peripheral nuclei compatible with hepatic steatosis (**C**). Arrows indicate many mononuclear cells, such as macrophages and lymphocytes (**G**,**H**). Grade of steatosis (**M**); ADA, adipocyte area (**N**); ADN, adipocyte number (**O**). † significant difference compared with NF; ‡ significant difference compared with NFMG; § significant difference compared with HF. Values are median to the 25th–75th percentiles (Kruskal–Wallis test and Tukey’s post hoc test, *p* ≤ 0.05), n = 8.

The liver tissue of the rats in the NF (Figure 3A) and NFMG (Figure 3B) groups presented a standard shape, with hepatocytes organized in cords without sinusoidal alterations. The rats in the HF (Figure 3C) and HFMG (Figure 3D) groups had hepatocytes with clear cytoplasm and peripheral nuclei, compatible with hepatic steatosis (black arrow), which varied in degree as follows: HF > HFMG (Figure 3F).

The rats’ colons in the NF (Figure 3E) and NFMG (Figure 3F) groups showed standard histology characterized by an epithelium filled with enterocytes with large amounts of goblet cells. However, the rats in the HF (Figure 3G) and HFMG (Figure 3H) groups presented an inflammatory process in the lamina propria of the organ with a richness of mononuclear cells, such as macrophages and lymphocytes (black arrow).

These results demonstrate that the highest degree of hepatic steatosis was directly associated with an accumulation of hepatic cholesterol (*p* = 0.85; *p* ≤ 0.001), increased malondialdehyde in the liver (*p* = 0.65; *p* ≤ 0.01) and lower fecal total bile acid excretion (*p* = −0.58; *p* ≤ 0.05). Furthermore, hepatic steatosis was positively correlated with the serum ALT levels (*p* = 0.73; *p* ≤ 0.001), AST (*p* = 0.77; *p* ≤ 0.001), triacylglycerides (*p* = 0.77; *p* ≤ 0.001), total cholesterol (*p* = 0.77; *p* ≤ 0.001), LDL (*p* = 0.67; *p* ≤ 0.001), VLDL (*p* = 0.62; *p* ≤ 0.05) and malondialdehyde (*p* = 0.57; *p* ≤ 0.05), and it was negatively with the serum HDL (*p* = −0.65; *p* ≤ 0.001) (Figure S3).

Histological (Figure 3I–L) and morphometric (Figure 3N,O) analyses of the adipose tissue revealed that the high-fat diet increased the area and number of adipocytes in the HF, but there was a significant reduction in these parameters in the HFMG rats (*p* ≤ 0.05), even with similar parameters to rats fed a normal-fat diet (NF and NFMG).

## 4. Discussion

Evidence indicates that bioactive compounds present in citrus fruits, such as mangaba, acerola [2,5,8], carambola [39], araçá [40], and jaboticaba [41] (among others), have health benefits, including prevention or adjuvant treatment of cardiovascular diseases [11,12,13]. The main results showed that the phenolic compounds, vitamin C, dietary fiber, oligosaccharides and carotenoids (Table S1) present in mangaba powder can play an important role as an adjuvant treatment in rats fed a high-fat diet. Rats fed a high-fat diet have been a widely used animal model for demonstrating the effects of different foods on lipid metabolism, liver health and antioxidant status, which may be an important tool for future translational studies [5,6,8,9,10,36].

### 4.1. Somatic Parameters, Monitoring Body Weight and Dietary, Lipid and Energy Intake

The HF rats had lower dietary and energy intake, but higher lipid consumption. The elevated lipid content of the high-fat diet contributes to a high energy density, reducing food and energy intake [8,9]. Similar results have already been observed in rats fed a high-fat diet for five weeks [5,6]. Some studies report that dietary fat consumption is associated with increased excretions of the cholecystokinin hormones, glucagon-like peptide 1 and peptide YY by intestinal mucosa cells, which act on satiety and decrease food intake [5,6,9]. Furthermore, high-fat diets increase enterostatin levels –a peptide that participates in the fat digestion process—also causing physiological satiety and reducing caloric intake [42].

It is important to highlight that despite the high lipid consumption, the rats in the HF and HFMG groups did not become obese during the experimental period, as they had a body mass index below 0.68 [29], Lee index below 0.30 [43] and adiposity index below 6.3% for adult rats [30]. In turn, the increase in the liver and adipose tissue weight of rats fed a high-fat diet is related to fat accumulation [2,6], as evidenced by the histological analyses discussed below.

### 4.2. Effect of Mangaba Powder on Lipid Parameters, Indices Related to Cardiovascular Health and Aminotransferases

The reduction in the total cholesterol levels in the NFMG and HFMG groups; reduction in the triacylglycerides, LDL, and VLDL levels in the NFMG group; and the increase in HDL levels, reflected in the lower risk indexes related to cardiovascular health in rats treated with mangaba powder (NFMG and HFMG), together showing the lipid-lowering effect of this product.

The negative correlation among HDL and the total cholesterol, atherogenic index, coronary risk index, and cardiovascular risk index, reinforces the role of HDL in reducing the risk of developing atherosclerosis, since this lipoprotein is responsible for the reverse transport of cholesterol to the liver [44]. In addition, HDL was inversely related to the circulating triacylglyceride levels, which may also contribute to preventing coronary heart disease [45,46].

The lipid-lowering effect of mangaba powder is possibly related to its composition of bioactive compounds, especially dietary fiber and phenolic compounds. These bioactive compounds improve triacylglyceride lipolysis for energy production and decrease fatty acid and triacylglyceride synthesis in the liver [8].

Dietary fiber, whether soluble or insoluble, such as those quantified in mangaba powder, sequesters cholesterol and increases the fecal excretion of lipids and bile acids, leading to the reduction of hepatic cholesterol absorption and production of new bile acids from endogenous cholesterol, which is consequently also related to a decrease in the risk of developing cardiovascular disease [47].

Additionally, there is evidence that oligosaccharides, kestose and raffinose, quantified for the first time in mangaba, serve as substrates for beneficial bacteria of the intestinal microbiota, participate in regulating lipid absorption in the intestine, and contribute to the reduced total cholesterol, triacylglycerides and phospholipid levels [8,48].

Among the major phenolic compounds in mangaba powder, chlorogenic acid can improve the lipid profile with a similar mechanism to statin drugs by inhibiting the 3-hydroxy-3-methyl-glutaryl CoA reductase enzyme, limiting the synthesis of endogenous cholesterol, which in turn influences a reduction in the blood cholesterol levels [47,49]. B1 and B2 procyanidins, also quantified in mangaba powder, are associated with serum LDL reduction, owing to the antioxidant mechanism that prevents lipid oxidation and limits the flow of atherogenic lipoproteins in the arterial wall. Furthermore, procyanidins stimulate the release of nitric oxide, a potent endothelial vasodilator that can reduce platelet aggregation—a mechanism involved in atherothrombosis [50].

The synergistic effect between the nutrients and/or phytochemicals contained in citrus fruits, such as mangaba, also contributes to lipid metabolism control [13]. An in vivo study found that the ascorbic acid, in association with flavonoids, improved the endothelial function of coronary and peripheral vessels by inhibiting the absorption of cholesterol and bile acids and increasing the catabolism of cholesterol in the liver [51].

Furthermore, increased levels of ALT and AST enzymes, seen in the HF group, are signs of diseases or damage to liver tissue and are commonly observed in individuals with NAFLD, in which their activities are increased by up to five times the upper limit of normality [52]. Importantly, the high concentration of liver enzymes resulting from NAFLD and underlying hepatic inflammation was associated with the development of cardiovascular disease in humans, since NAFLD is one of its main risk factors [53,54]. Nonetheless, mangaba powder significantly attenuates the changes in the AST concentrations in the HFMG group. In agreement with these results, chlorogenic acid reduced hepatic fat accumulation and serum AST and ALT values in C57BL/6J mice fed a high-fat diet [55]. This phenolic acid is a bioactive compound found in high quantities in mangaba fruit [11,12] and the mangaba powder used in our study.

### 4.3. Triacylglycerides, Total Cholesterol and Total Bile Acids in Feces and Liver

The final body weight of the rats had a significant negative correlation with the fecal triacylglycerides and a positive correlation with the hepatic triacylglycerides, which indicates that an increase in body weight may influence lower fecal triacylglycerides excretion and the triacylglycerides accumulation in the liver, as seen in the HF rats. However, mangaba powder attenuated this effect in the HFMG rats.

The fat content in the liver reflects the balance between various metabolic pathways involved in the synthesis and elimination of triacylglycerides, such as lipolysis in adipose tissue, lipogenesis, triacylglyceride esterification, fatty acid oxidation and lipoprotein synthesis/secretion in liver tissue [56].

The reduction of total cholesterol in the liver of the HFMG group may be related to the dietary fiber levels in mangaba powder, especially soluble ones. The gut microbiota ferments soluble fiber to produce short-chain fatty acids: acetic, propionic, and butyric acids. These short-chain fatty acids play a crucial role in lipid metabolism by reducing adipogenesis, lipogenesis and fat accumulation in cells [57,58]. Oligosaccharides are non-digestible carbohydrates that serve as substrates to produce short-chain fatty acids, particularly propionic acid. This acid benefits cholesterol metabolism by increasing intestinal IL-10 production and this interleukin regulates the expression of the principal intestinal cholesterol transporter named Niemann-Pick C1-like 1 [59].

A previous study has shown that flavonoids, including hesperidin, which are present in mangaba powder, inhibited lipogenesis and induced fatty acid oxidation in the liver, causing decreased serum cholesterol and triacylglycerides in rats fed a high-fat diet [60]. Other phenolic compounds quantified in mangaba powder, such as catechins and quercetin 3-glycoside, may also positively impact the lipid metabolism of rats fed a high-fat diet. Catechins inhibit lipid absorption in the intestine by interfering with micelle formation, emulsification, hydrolysis, solubilization, and inhibition of squalene oxidase (a key enzyme in the biosynthesis of hepatic cholesterol) [61]. In turn, quercetin 3-glycoside serves as a substrate for the intestinal microbiota, which by indirect action reduces the atherogenic lipid levels, such as cholesterol and lysophosphatidic acid molecular markers [62].

The high production of total bile acids in the liver in the NFMG and HFMG rats, and the non-observance of an increase in the fecal total bile acid excretion in these groups, may be related to balancing the synthesis and excretion of bile salts, which prevents both functional deficiencies and toxic accumulation of this substance in the body, which will depend on the animal’s ability to convert cholesterol into bile acids [63]. An increase in the bile acid concentration in the liver causes cholestasis (a pathophysiological process in which biliary drainage is deficient, causing liver damage) [64]. Significantly, bioactive compounds such as vitamin C and quercetin, present in mangaba powder, have been shown to exert hepatoprotective effects in rats with cholestatic liver [65].

Furthermore, the liver total bile acid levels were positively correlated with the total antioxidant capacity and negatively correlated with the hepatic malondialdehyde biomarker. This result shows that an elevated total antioxidant capacity in the liver could reduce lipid peroxidation and controllably increase total bile acid production in the organ [66].

### 4.4. Malondialdehyde and Total Antioxidant Capacity

The lower lipid peroxidation observed in the serum and liver of the HFMG rats may be attributed to the reaction of bioactive components in mangaba powder with free radicals. Among these compounds, ascorbic acid and carotenoids interact and stabilize superoxide radical anion, hydroperoxyl radical and hydroxyl radical, inhibiting the lipid peroxidation reactions [67].

The in vivo antioxidant capacity of mangaba powder can also be associated with the synergistic effects of bioactive compounds such as a combination of polyphenols and vitamin C or flavonoids and proteins, which increased the effectiveness of the in vitro antioxidant effect [67]. It is added that mangaba powder presented high antioxidant activity in vitro, being able to inhibit DPPH and ABTS radicals. Furthermore, the antioxidant activity of freeze-dried fruits can be superior to the antioxidant activity of fresh fruits, and this is because the antioxidant compounds are concentrated after the dehydration process [68].

### 4.5. Effects of Mangaba Powder Treatment on Liver, Intestine and Adipose Tissue Histology

The consumption of mangaba powder was able to attenuate the hepatic steatosis caused by the high-fat diet in rats; however, it was not possible to reverse the inflammatory process in the colon. Consumption of phenolic compounds was associated with a reduction in the number and area of adipocytes in rats fed a high-fat diet, and this action may be related to the decrease of the fatty acid synthase activity (the main enzyme in fatty acid biosynthesis) and synthesis of triacylglycerides and cholesterol in the liver [2,69]. This potential mechanism of action by mangaba powder may also explain the low serum total cholesterol and LDL levels in the HFMG and NFMG rats. In addition, the amount of vitamin C in mangaba powder may favor these results, since vitamin C administered alone to mice reduced the size of adipocytes and contributed to lipogenesis inhibition [70].

It is worth remembering that higher levels of circulating lipids, seen in HG rats, favor accumulating liver fat and abdominal lipogenesis, with the opposite being true in this bidirectional pathway. Thus, dietary lipids influence the liver’s function as a central regulator of lipid homeostasis [71].

On the other hand, the hepatoprotective, lipid-lowering and antioxidant effects demonstrated by mangaba powder are compatible with the beneficial effects of administering nutraceuticals, phytotherapeutics and fruit extracts rich in polyphenols, which have also been evaluated in experimental animal models fed a high-fat diet. For example, the consumption of Tucum-do-Pantanal (*Bactris setosa* Mart) and Tarumã-do-Cerrado (*Vitex cymosa Bertero* ex Spreng) fruit extracts reduced weight gain, decreased adiposity, and prevented NAFLD in mice that consumed a high-fat diet [72]. The administration of ginger extract (*Zingiber officinale* Roscoe) and resveratrol, recognized for their antioxidant properties in vitro and in vivo, minimized hepatic steatosis in rodents fed a high-fat diet [73,74].

As a limitation of the present study, we observed that other doses of the mangaba powder could have been administered to rats fed a high-fat diet for more time to establish a dose–response curve. Furthermore, other studies that molecularly investigate the mechanisms of action of mangaba powder are still needed. However, regular consumption of mangaba powder, whether by rats fed a normal- (NFMG) or high-fat diet (HFMG), improved the somatic parameters, lipid metabolism, liver health, and oxidative parameters in the rats.

Our results should be analyzed with caution when it comes to the translation from rodents to humans, since although studies with animal models are excellent tools for directing clinical research, all the phases of translational research (T0–T4) need to be performed, as well as respecting the similarities and differences in physiological and behavioral characteristics between humans and rodents [75]. Nonetheless, our results reinforce the need for translational studies with a healthy population or with cardiovascular diseases such as dyslipidemia and to be carried out based on the amount of product administered to rats, which is equivalent to 3.89 g for an individual weighing 60 kg [76]. The results can encourage the exploitation this fruit and the development of a production chain focused on the food and supplement industry.

## 5. Conclusions

The mangaba powder had a lipid-lowering effect and protected the liver from damage caused by consuming the high-fat diet, which was demonstrated by the improvement in the lipid profile, the increase in the total antioxidant capacity and the minimized lipid peroxidation at the hepatic and systemic levels, in addition to a reduction in the AST levels and degree of hepatic steatosis. Regular consumption of mangaba powder also improved the somatic parameters, mainly related to adiposity in rats fed a high-fat diet.

The beneficial effects shown herein are possibly related to the bioactive compounds present in the product, mainly dietary fiber, oligosaccharides, phenolic compounds and vitamin C, which may have acted in additively and synergistically in metabolizing dietary lipids, promoting liver health and increasing in vivo antioxidant capacity. Future studies focusing on investigating the mechanisms behind the effects reported in this pioneering study could reinforce the understanding the promising metabolic effects of mangaba powder.

## Figures and Tables

**Table 1:** Somatic parameters of rats fed normal- or high-fat diets and treated or not with mangaba powder.

Parameters	Groups				
	NF	NFMG	HF	HFMG	*p*
FBW (g)	360.00 (338.37–403.65)	347.50 (330.94–357.81)	380.00 (352.21–417.79) ‡	367.50 (336.25–381.25)	0.029
BL (cm)	23. 50 (21.12–23.62)	25.00 (23.00–26.25)	24.01 (23.00–24.50)	24.03 (23.50–24.87)	n.s.
WC (cm)	17.25 (15.87–19.15)	17.00 (16.00–18.00)	17.50 (17.50–18.00)	16.75 (14.62–18.00)	n.s.
CC (cm)	15.25 (14.12–15.85)	15.00 (14.00–15.00)	15.00 (15.00–16.00)	15.75 (15.00–17.37)	n.s.
BMI (g/cm^2^)	0.62 (0.61–0.63)	0.56 (0.50–0.58) †	0.65 (0.61–0.66) ‡	0.60 (0.57–0.63) ‡§	<0.001
Lee index	0.30 (0.29–030)	0.28 (0.27–0.28) †	0.30 (0.29–0.31) ‡	0.29 (0.29–0.30) ‡	<0.001
ADI (%)	1.85 (1.76–2.93)	2.69 (2.48–2.83)	3.82 (3.76–3.92) †‡	3.28 (2.69–3.80) †‡	0.004
Liver weight (g)	3.53 (3.32–3.86)	3.47 (3.35–3.72)	5.41 (4.98–5.90) †‡	5.38 (4.31–5.68) †‡	<0.001
Adipose tissue weight (g)	1.71 (1.32–2.92)	2.61 (2.09–2.69)	3.86 (3.59–4.09) †‡	3.28 (2.66–3.93) †‡	0.029
Bowel weight (g)	4.33 (3.91–4.82)	4.53 (3.98–5.40)	3.29 (3.02–3.94) ‡	3.46 (3.14–4.07) †‡	0.016

NF: rats fed a normal-fat diet (n = 8); NFMG: rats fed a normal-fat diet with administration mangaba (n = 8); HF: rats fed a high-fat diet (n = 8); HFMG: rats fed a high-fat diet with administration mangaba (n = 8). BL, body length; FBW, final body weight; WC, waist circumference; CC, chest circumference; ADI, adiposity index; BMI, body mass index. † significant difference compared with NF; ‡ significant difference compared with NFMG; § significant difference compared with HF. n.s., not significant. Values are median to the 25th–75th percentiles (Kruskal–Wallis test and Tukey’s post hoc test, *p* ≤ 0.05), n = 8.

**Table 2:** Triacylglycerides, total cholesterol and total bile acids in feces and liver of rats fed normal- or high-fat diets and treated or not with mangaba powder.

Parameters	Groups				
	NF	NFMG	HF	HFMG	*p*
**Feces**					
TG (mg/g)	10.69 (9.49–11.89)	12.45 (11.80–13.10)	9.60 (9.30–9.90) ‡	16.33 (15.51–17.16) §	0.027
TC (mg/g)	3.38 (3.37–3.38)	4.44 (4.25–4.63)	4.84 (4.73–4.95) †	4.62 (3.37–4.62)	0.044
TBA (mg/g)	3.81 (3.75–3.86)	3.22 (2.96–3.48)	2.91 (2.85–2.98) †	2.89 (2.85–3.39)	0.040
**Liver**					
TG (mg/g)	65.70 (59.07–72.35)	65.94 (60.76–71.12)	82.79 (78.22–87.35) †‡	65.94 (60.76–71.12) §	n.s.
TC (mg/g)	10.91 (10.18–11.64)	10.58 (10.11–11.07) †	14.70 (14.53–14.87) †‡	11.72 (10.87–12.56) §	0.015
TBA (mg/g)	3.31 (3.04–3.84)	5.56 (4.58–7.00) †	2.94 (2.63–3.33) ‡	13.26 (12.80–13.83) †‡§	0.003

NF: rats fed a normal-fat diet (n = 8); NFMG: rats fed a normal-fat diet with administration mangaba (n = 8); HF: rats fed a high-fat diet (n = 8); HFMG: rats fed a high-fat diet with administration mangaba (n = 8). TG, triacylglycerides; TC, total cholesterol; TBA, total bile acids. † significant difference compared with NF; ‡ significant difference compared with NFMG; § significant difference compared with HF. n.s., not significant. Values are median to the 25th–75th percentiles (Kruskal–Wallis test and Tukey’s post hoc test, *p* ≤ 0.05), n = 8.

**Table 3:** Lipid peroxidation and total antioxidant capacity in serum and liver of rats fed normal- or high-fat diets and treated with mangaba powder.

Parameters	Groups				
	NF	NFMG	HF	HFMG	*p*
**Serum**					
MDA (µmol/L)	4.55 (4.38–5.07)	5.12 (4.99–6.59)	8.19 (7.48–8.71) †‡	5.79 (5.18–6.11) †§	0.005
TAC (µmol/L)	15.43 (11.84–19.02)	21.34 (16.54–25.00) †	18.02 (3.95–22.09)	26.30 (19.48–28.47) †	0.019
**Liver**					
MDA (µmol/g)	3.14 (1.44–3.66)	2.67 (2.59–2.87)	8.81 (6.42–9.86) †‡	2.77 (2.41–2.85) §	0.029
TAC (µmol/L)	30.38 (25.62–35.16)	78.53 (77.48–81.76) †	21.91 (17.13–25.70) ‡	93.55 (89.02–94.11) †§	<0.001

NF: rats fed a normal-fat diet (n = 8); NFMG: rats fed a normal-fat diet with administration mangaba (n = 8); HF: rats fed a high-fat diet (n = 8); HFMG: rats fed a high-fat diet with administration mangaba (n = 8). MDA, malondialdehyde; TAC, total antioxidant capacity. † significant difference compared with NF; ‡ significant difference compared with NFMG; § significant difference compared with HF. Values are median to the 25th–75th percentiles (Kruskal–Wallis test and Tukey’s post hoc test, *p* ≤ 0.05), n = 8.

## Data Availability

The original contributions presented in the study are included in the article/Supplementary Materials; further inquiries can be directed to the corresponding author.

## Supplementary Materials

The following supporting information can be downloaded at https://www.mdpi.com/article/10.3390/foods13233773/s1, Table S1: Bioactive components and antioxidant activity of mangaba powder; Table S2: Composition of the normal- and high-fat diets consumed by Wistar rats treated or not with mangaba powder; Table S3: Fatty acid composition of the normal- and high-fat diets consumed by Wistar rats treated or not with mangaba powder; Figure S1: Dietary intake (A), energy intake (B), lipid intake (C), body weight (D) and areas under the curve (E-H) in the dyslipidemia induction; Figure S2: Lipid profile to diagnostic dyslipidemia induction in Wistar rats (A-E); Figure S3: Spearman correlation matrix considering parameters of rats fed normal- or high-fat diets and treated or not with mangaba powder.
