# Correction: Silva et al. Hepatoprotective, Lipid-Lowering and Antioxidant Effects of Mangaba Powder (*Hancornia speciosa*) Administered to Rats Fed a High-Fat Diet. *Foods* 2024, *13*, 3773

**DOI:** 10.3390/foods14111993

**Published:** 2025-06-05

**Authors:** Bernadete de Lourdes de Araújo Silva, Margarida Angélica da Silva Vasconcelos, Kamila Sabino Batista, Marcos dos Santos Lima, Fabiane Rabelo da Costa Batista, Hassler Clementino Cavalcante, Lydiane de Lima Tavares Toscano, Alexandre Sérgio Silva, Aline Barbosa D’Oliveira, Adriano Francisco Alves, Jailane de Souza Aquino

**Affiliations:** 1 Nutrition Department, Universidade Federal de Pernambuco, Recife 50670-901, Brazil; bernadete.araujo2@academico.ufpb.br (B.d.L.d.A.S.); margarida.vasconcelos@ufpe.br (M.A.d.S.V.); 2 Nutrition Department, Universidade Federal da Paraíba, João Pessoa 58051-900, Brazil; hasslerrcavalcante@gmail.com (H.C.C.); lyditavares@hotmail.com (L.d.L.T.T.); alexandre.sergio@ccs.ufpb.br (A.S.S.); 3 Experimental Nutrition Laboratory, Universidade Federal da Paraíba, João Pessoa 58051-900, Brazil; kamila.sabino@outlook.com (K.S.B.); allineolliveira99@gmail.com (A.B.D.); 4 Instituto Nacional do Semiárido, Campina Grande 58434-700, Brazil; fabiane.costa@insa.gov.br; 5 Department of Food Technology, Federal Institute of the Sertão of Pernambuco (IFSertãoPE), Petrolina 56316-686, Brazil; marcos.santos@ifsertao-pe.edu.br; 6 Department of Physiology and Pathology, Universidade Federal da Paraíba, João Pessoa 58051-900, Brazil; adrianofalves@ccs.ufpb.br

There was an error in the original publication [1]. A correction has been made to add an author; the updated author is as follows:

**Addition of an Author**

Marcos dos Santos Lima was not included as an author in the original publication. The corrected Author Contributions statement appears here.

Conceptualization, formal analyses, investigation, resources, writing—original draft, writing—review and editing.

The authors state that the scientific conclusions are unaffected. This correction was approved by the Academic Editor. The original publication has also been updated.
