# Supplementary material for: Hepatoprotective, Lipid-Lowering and Antioxidant Effects of Mangaba Powder (Hancornia speciosa) Administered to Rats Fed a High-Fat Diet
Source: Foods. 2024 Nov 25;13(23):3773. doi: 10.3390/foods13233773 (PMC11640672; doi:10.3390/foods13233773)
Supplement: Supplementary file 1 [file foods-13-03773-s001.zip › foods-3315668-supplementary.pdf]

Supplementary material

Table S1: Bioactive components and antioxidant activity of mangaba powder

**Bioactive components and antioxidant activity**	**Mangaba powder**
TPC (mg GAE/100g)	1524.90 ± 32.44
Total carotenoids (mg/100g)	0.10 ± 0.03
Vitamin C (mg/100g)	140.20 ± 3.09
**Phenolic profile** (mg/100 g)	
Hesperidin	0.35 ± 0.02
Procyanidin B1	2.84 ± 0.00
Procyanidin B2	2.30 ± 0.03
Catechin	0.44 ± 0.09
Epigallocatechin gallate	0.10 ± 0.01
Quercetin 3-glucoside	2.24 ± 0.08
Kaempferol 3-glucoside	0.03 ± 0.03
Rutin	0.03 ± 0.01
Chlorogenic acid	2.87 ± 0.05
Trans-resveratrol	0.32 ± 0.04
**Antioxidant activity** (µmol Trolox /100g)	
DPPH	3548.67 ± 8.50
ABTS	1653.82 ± 45.91
**Oligossacharides** (mg/100g)	
Kestose	0.15 ± 0.02
Raffinose	17.48 ± 0.46
**Dietary fibre** (g/100 g)	
TDF	4.10 ± 0.14
SDF	1.55 ± 0.06
IDF	2.55 ± 0.12

Means and standard deviation (±) three repetitions (n=3). Results expressd on dry basis. TDF, Total dietary fibre; SDF, Soluble dietary fibre; IDF, Insoluble dietary fibre; TPC total phenolic compounds.

Table S2: Composition of the normal- and high-fat diets consumed by Wistar rats treated or not with mangaba powder.

Ingredients (g/100g)	Diets	
	AIN-93M*	Dyslipidaemic**
Milk casein	14.00	16.50
Maize starch	47.00	36.45
Dextrinized starch	15.50	15.50
Sucrose	10.00	6.00
Fibre	5.00	5.00
Soy oil	4.00	3.00
Animal fat (lard)	-	6.00
Non-hydrolyzed vegetable fat	-	5.00
Sigma cholic acid	-	0.50
Sigma cholesterol	-	1.00
Mineral mix AIN -93M	3.50	3.50
Vitamin mix	1.00	1,00
L-cysteine	0.18	0.30
Hill bitartrate	0.25	0.25
t-BHQ***	0.008	0.014
Calories (kcal/g)	3.82	4.25
Carbohydrates (kcal %)	75.77	54.54
Proteins (kcal %)	14.82	15.81
Lipids (kcal %)	9.40	29.64

*Adapted from Reeves et al., (1993). **Rhoster Industria and Comércio Ltda.***: t-BHQ, tert-butylhydroquinone.

Table S3: Fatty acid composition of the normal- and high-fat diets consumed by Wistar rats treated or not with mangaba powder

**Fatty Acids (g /100g)**	**Diets**	
	**AIN-93M**	**Dyslipidaemic**
Myristic acid C 14:0	0.50 ± 0.06	-
Palmitic acid C16:0	16.41 ± 0.10	33.23 ± 0.22
Stearic acid C18:0	11.22 ± 0.45	5.98 ± 0.05
Arachidic acid C20:0	0.21 ± 0.13	-
**Saturated Fatty Acids (SFA g /100g)**	**28.34**	**39.21**
Palmitoleic acid C16:1 n7	0.79 ± 0.07	-
Heptadecenoic acid C17:1 n7	0.21 ± 0.03	-
Oleic acid C18:1 n9	29.65 ± 0.12	35.14 ± 0.11
**Monounsaturated Fatty Acids (MUFA g /100g)**	**30.95**	**35.14**
Elaidic acid C18:1 n9t	3.55 ± 0.02	1.77 ± 0.12
Linoleic acid C18:2 n6	35.89 ± 0.35	20.16 ± 0.37
Alpha-linolenic acid C18:3n3	0.76 ± 0.05	3.72 ± 0.20
Cis-11-Eicosenoic acid C20:1n9	0.51 ± 0.09	-
Eicosapentaenoic acid C20:5 n3 (EPA)	-	-
Docosahexaenoic acid C22:6n3 (DHA)	-	-
**Polyunsaturated Fatty Acids (PUFA g /100g)**	**40.71**	**25.65**

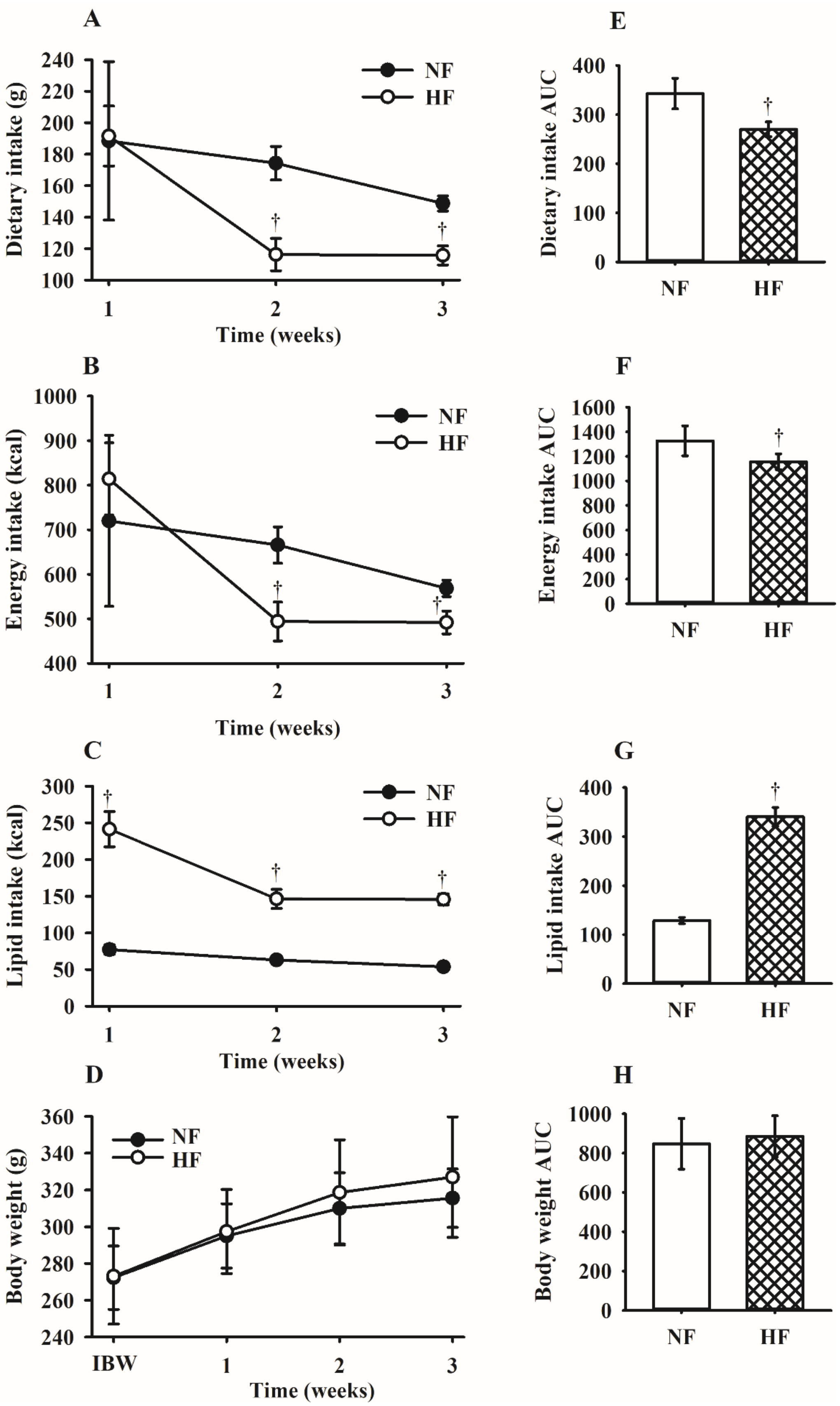

Figure S1: Dietary intake (A), energy intake (B), lipid intake (C), body weight (D) and areas under the curve (E-H) in the dyslipidemia induction. AUC, areas under the curve; HG, healthy group; DG, dyslipidaemic group; IBW, initial body weight; † significant difference compared with HG. Values are median to the 25th - 75th percentiles (Mann-Whitney U test, $P < 0.05$), n = 16.

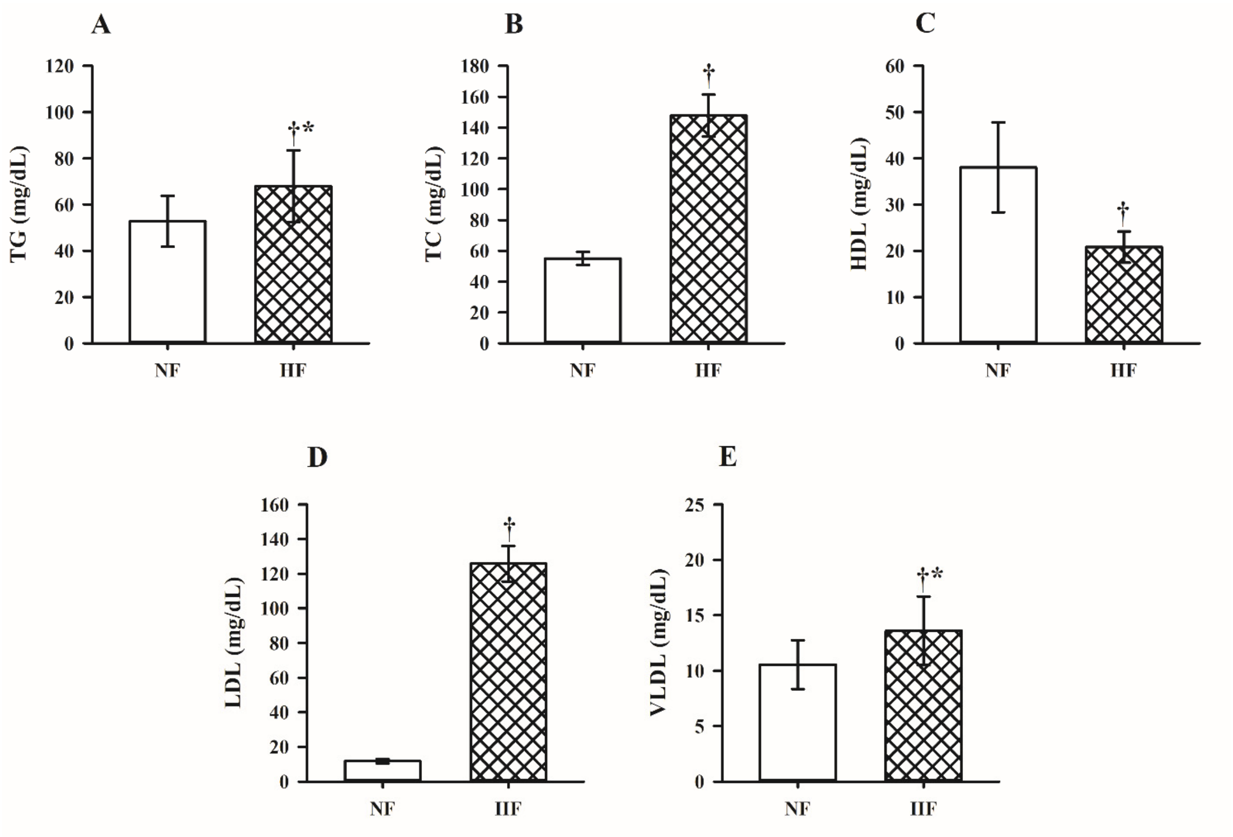

Figure S2: Lipid profile to diagnostic dyslipidemia induction in Wistar rats (A-E). HG, healthy group; DG, dyslipidaemic group; TC, total cholesterol; TG, triglycerides; LDL, low density lipoprotein; VLDL, very low-density lipoprotein; HDL, high density lipoprotein. † significant difference compared with HG. Values are median to the 25th - 75th percentiles (Mann-Whitney U test, $P < 0.05$), $n = 16$.

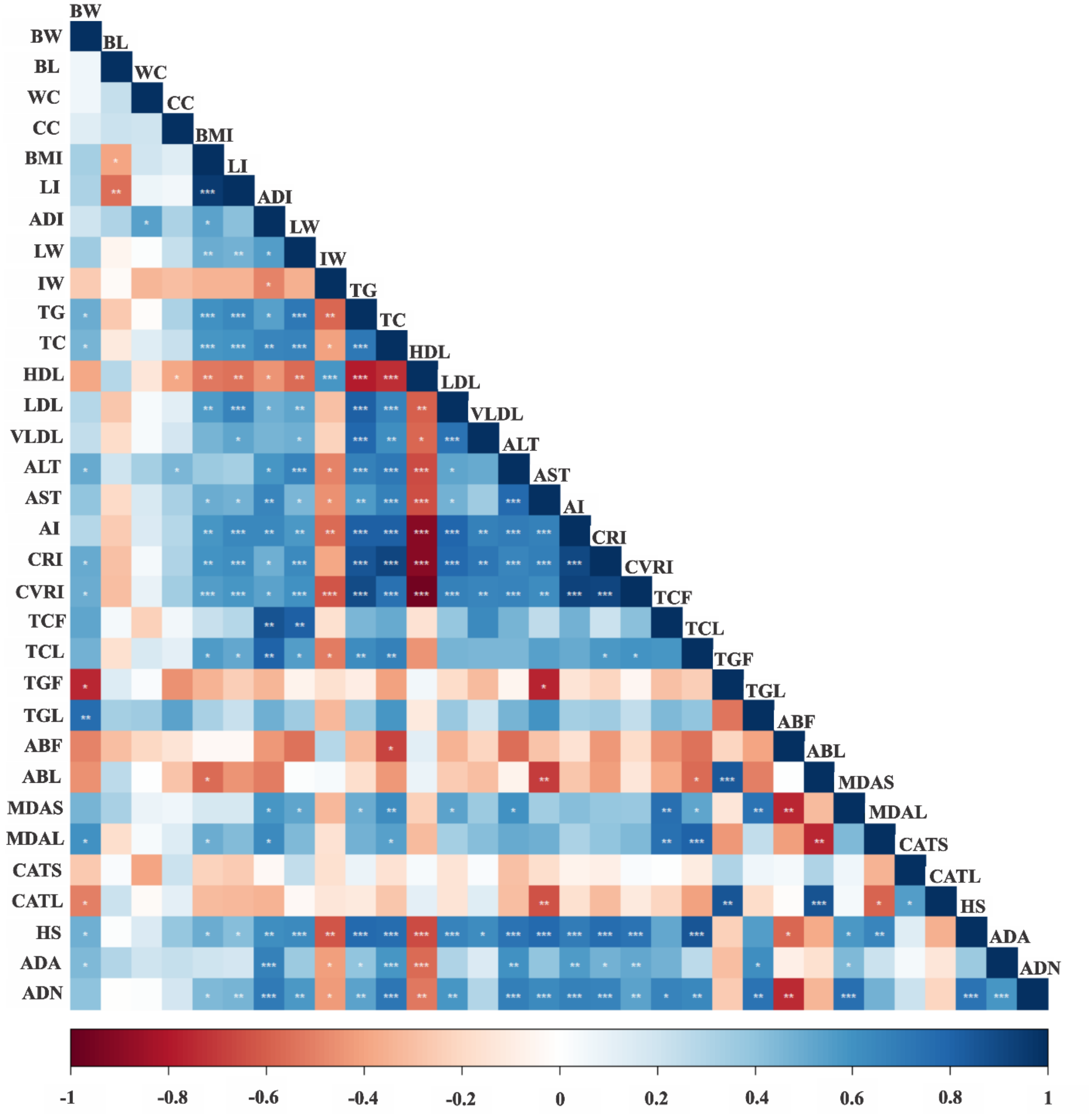

Figure S3: Spearman correlation matrix considering parameters of rats fed normal- or high-fat diets and treated or not with mangaba powder**.** * P <0.05; ** P <0.01; *** P <0.001. FBW, final body weight; BL, body length; WC, waist circumference; CC, chest circumference; BMI, body mass index; LI, lee index; BMI, body mass index; LI, Lee index; ADI, adiposity index; LW, liver weight; IW, intestine weight; TG, triglycerides; TC, total cholesterol; TCF, total cholesterol faecal; TGL, triglycerides liver; LDL, low density lipoprotein; VLDL, very low density lipoprotein; HDL, high density lipoprotein; AI, atherogenic index; CRI, coronary risk index; CVRI, cardiovascular risk index; TCF, total cholesterol faecal; TCL, total cholesterol liver; ABF, bile acids faecal; ABL, bile acids liver; MDAS, serum malondialdehyde; MDAL, liver malondialdehyde; CATS, total serum antioxidant capacity; CATL, total liver antioxidant capacity; HS, hepatic steatosis; ADA, adipocity area; ADN adipocity number; AST, aspartate aminotransferase; ALT, alanine aminotransferase.
